# Supplementary material for: Interplay between IL-10, IFN-γ, IL-17A and PD-1 Expressing EBNA1-Specific CD4+ and CD8+ T Cell Responses in the Etiologic Pathway to Endemic Burkitt Lymphoma
Source: Cancers (Basel). 2021 Oct 27;13(21):5375. doi: 10.3390/cancers13215375 (PMC8582526; doi:10.3390/cancers13215375)
Supplement: Supplementary file 1 [file cancers-13-05375-s001.zip › cancers-1403519-supplementary.pdf]

cancers

MDPI

Supplementary Material

Interplay between IL-10, IFN-γ, IL-17A and PD-1 Expressing EBNA1-Specific CD4+ and CD8+ T Cell Responses in the Etiologic Pathway to Endemic Burkitt Lymphoma

Catherine S. Forconi, David H. Mulama, Priya Saikumar Lakshmi, Joslyn Foley Juliana A. Otieno, Jonathan D. Kurtis, Leslie J. Berg, John M. Ong'echa, Christian Münz and Ann M. Moormann

Table S1. Population characteristics.

	**Nandi (*n* = 20)**	**Kisumu (*n* = 19)**	**eBL (*n* = 19)**	**Nandi vs Kisumu (*p*)**	**Nandi vs eBL (*p*)**	**Kisumu vs eBL (*p*)**
Age[A]:	8 [2–14.6]	6.85 [1–14.8]	8.3 [3.3–11.90]	0.45 [B]	0.58 [B]	0.25 [B]
Sex (% of male)	57%	66%	63%	0.54 [C]	0.69 [C]	0.82 [C]
EBV load (copies per µg of human DNA)[A]	0 [0–192]	0 [0–320]	10,773 [0–183,568]	0.87 [B]	<0.0001 [B]	<0.0001 [B]
EBV seropositivity (%)	100%	100%	100%	>0.99 [B]	>0.99 [B]	>0.99 [B]
Serology expressed in Median Fluorescence Intensity (MFI)[A]						
EBNA1	15,861 [53–23,657]	12,794 [8,700–26,657]	9,659 [66–18,602]	0.93 [B]	0.04 [B]	0.04 [B]
VCA	17,380 [72–23,877]	10,465 [7,527–23,530]	17,714 [6,289–25,711]	0.13 [B]	0.61 [B]	0.24 [B]
MSP-1	2,535 [330–8,592]	6,542 [2,000–15,995]	6,568 [1,205–20,057]	0.01 [B]	0.007 [B]	>0.99 [B]
AMA1	211 [116–10,362]	18,118 [2,251–28,423]	19,849 [644–31689]	<0.0001 [B]	<0.0001 [B]	0.71 [B]
Ratio $CD4^+/CD8^+$ T cells[A]	2.38 [1.14–6.19]	2.75 [1.50–9.93]	2.75 [1.03–4.56]	0.06 [B]	0.57 [B]	0.22 [B]
% $CD14^-CD19^-CD3^+$ cells[A]	86 [59–98]	95 [88–98]	94 [73–98]	0.002 [B]	0.01 [B]	0.30 [B]

[A] Median [min–max] are represented; [B] *p* value from Mann Whitney statistical test; [C] *p* value from χ^2 statistical test

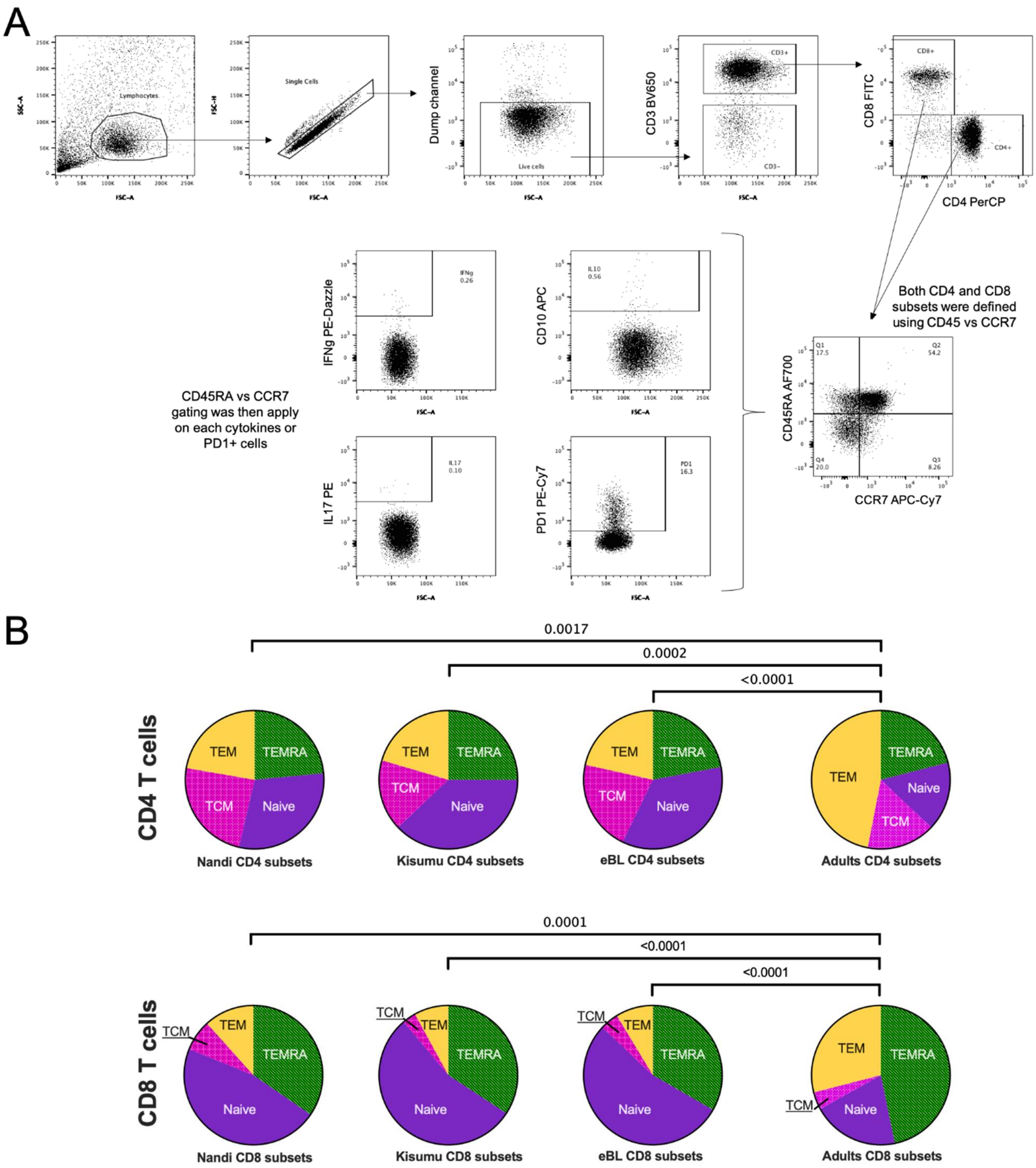

Figure S1. Flow cytometry gating strategy and T cell subsets. (**A**) Compensation and FMO control tubes were made for each experiment and used to design the compensation matrix and to determine our gating. First, lymphocytes were identified by SSC-A vs. FSC-A cytoplots. After selecting single cells (FSC-A vs. FSC-H), a dump channel allowed us to select only $CD14^-CD19^-$ live cells. Then, a clear $CD3^+$ gate isolated T-cells that were differentiated by CD8 vs. CD4 expression. CD45RA and CCR7 expression defined the following $CD4^+$ and $CD8^+$ T cell subsets: T_{EMRA} ($CD45RA^+CCR7^-$), $T_{Naive\text{-}like}$ ($CD45RA^+CCR7^+$), T_{CM} ($CD45RA^-CCR7^+$) and T_{EM} ($CD45RA^-CCR7^-$). We also evaluated the percentage of cytokines and/or PD-1 expressing $CD4^+$ and $CD8^+$ T cells and identified their subsets of origin based on CD45RA/CCR7 expression. (**B**) CD4 and CD8 T cell subsets were defined by CD45RA and CCR7 expression: T_{EMRA} (green, $CD45RA^+CCR7^-$), $T_{NAIVE\text{-}LIKE}$ (purple, $CD45RA^+CCR7^+$), T_{CM} (pink, $CD45RA^-CCR7^+$) and T_{EM} (yellow, $CD45RA^-CCR7^-$). The mean proportion of each subset is represented (pie slice). χ^2 test was applied and p values are indicated.

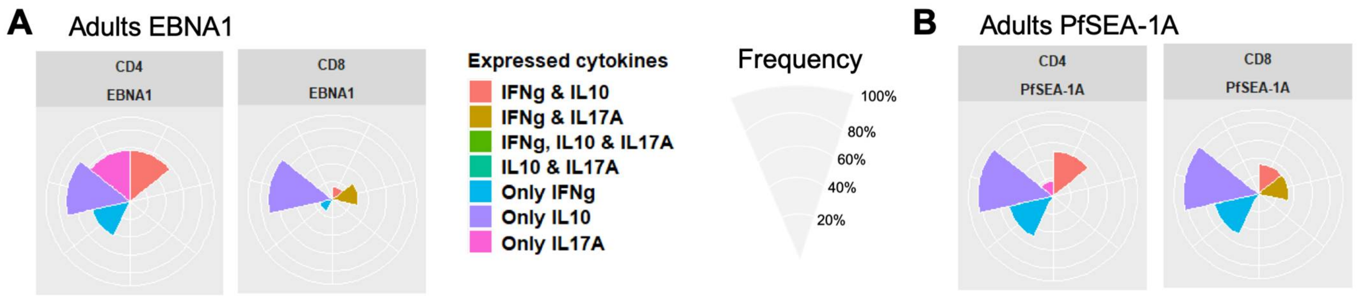

Figure S2. Adult T cell cytokine profiles in response to EBNA1 and *Pf*SEA-1A. Radar/flower plots represent the frequency (from 0 to 100%) of Kenyan adults who express cytokines from CD4⁺ and/or CD8⁺ after (A) EBNA1 or (B) *Pf*SEA-1A stimulation. Each petal represents a different combination of cytokine (IFN-γ, IL-10, IL-17A) expression

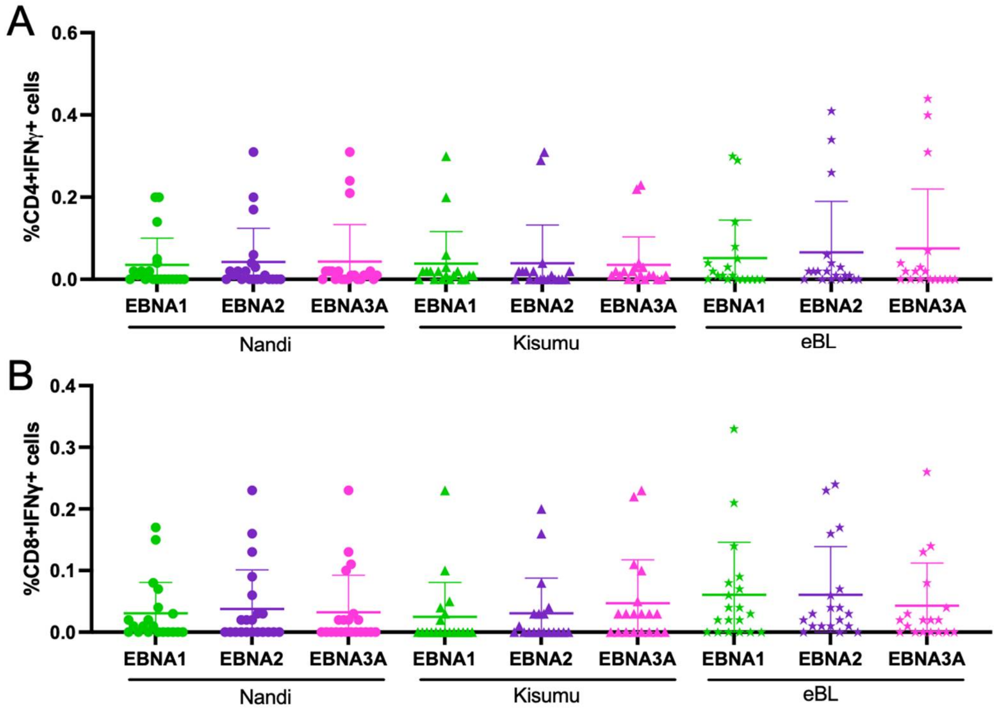

Figure S3. Comparison of IFN-γ^+ T cell responses between EBNA1, EBNA2 and EBNA3A stimulation for each group of children. Dot plots of mean and standard deviation (SD) for (**A**) $CD4^+$ and (**B**) $CD8^+$ T cells expressing IFN-γ^+ after EBNA1 (green), EBNA2 (purple), and EBNA3A (pink) stimulation across our groups of children Nandi (round), Kisumu (triangle), and eBL (star). No p values were significant ($p < 0.05$).

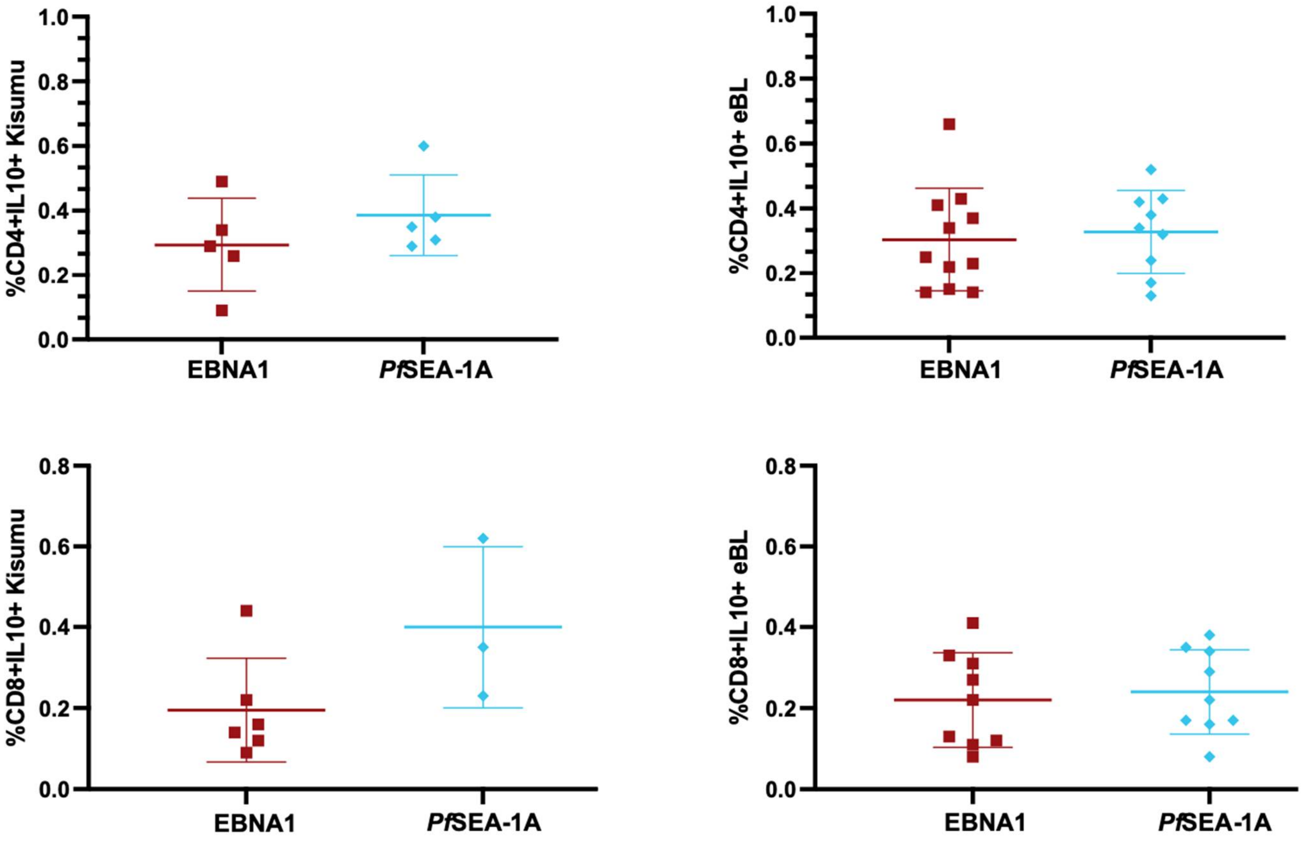

Figure S4. No differences were observed in IL-10 expression between EBNA1 and *Pf*SEA-1A for Kisumu or eBL children. IL-10⁺ CD4⁺ and CD8⁺ T cells responses after EBNA1 (red squares) and *Pf*SEA-1A (blue lozenges) stimulation, mean and SD are represented. No *p* values were significant ($p < 0.05$).

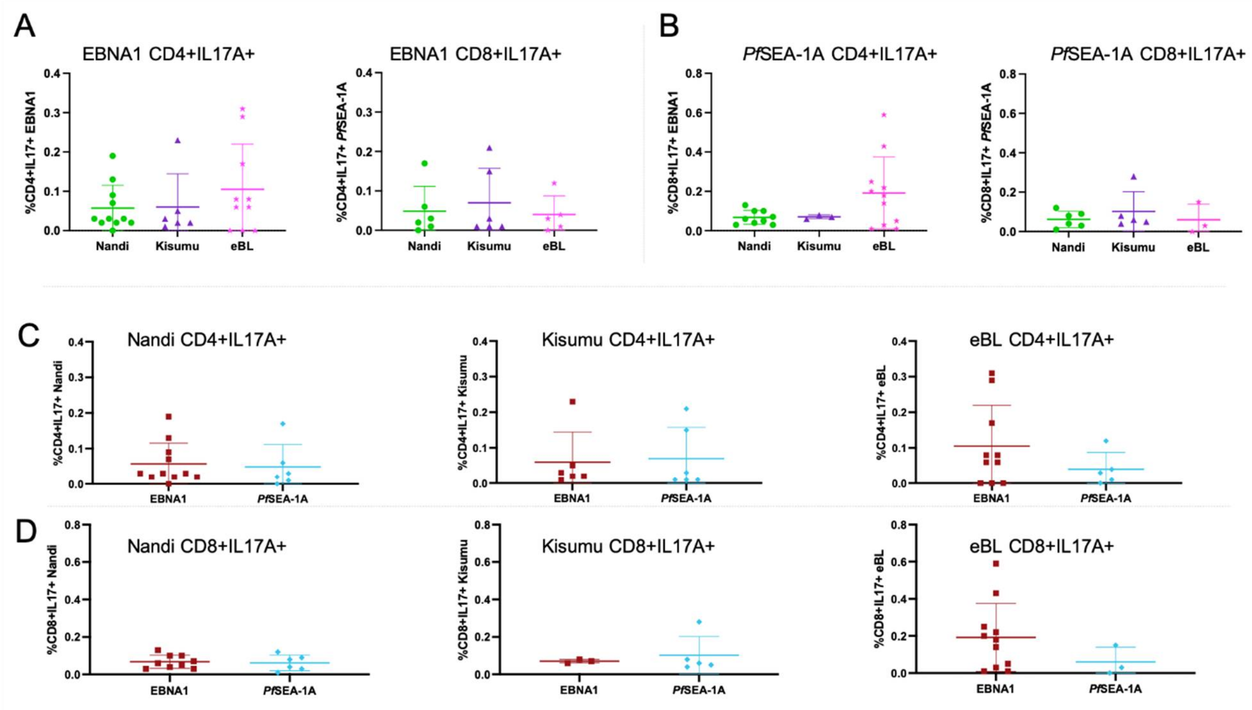

Figure S5. IL-17A responses to EBNA1 and *Pf*SEA-1A from CD4⁺ and CD8⁺ T cells within each group of children. Total CD4⁺ and total CD8⁺ T cells expressing IL-17A across the different groups of children: Nandi (green round), Kisumu (purple triangles) and eBL (pink stars) after (**A**) EBNA1 and (**B**) *Pf*SEA-1A stimulation. (**C**) Comparative dot plot of IL-

17A⁺CD4⁺ T cells response between EBNA1 (red squares) and *Pf*SEA-1A (blue lozenges) within Nandi (left), Kisumu (middle), and eBL (right) children. (**D**) Comparative dot plot of IL-17A⁺CD8⁺ T cells response between EBNA1 (red squares) and *Pf*SEA-1A (blue lozenges) within Nandi (left), Kisumu (middle), and eBL (right) children. Mean and SD are represented. No *p* values were significant ($p<0.05$).

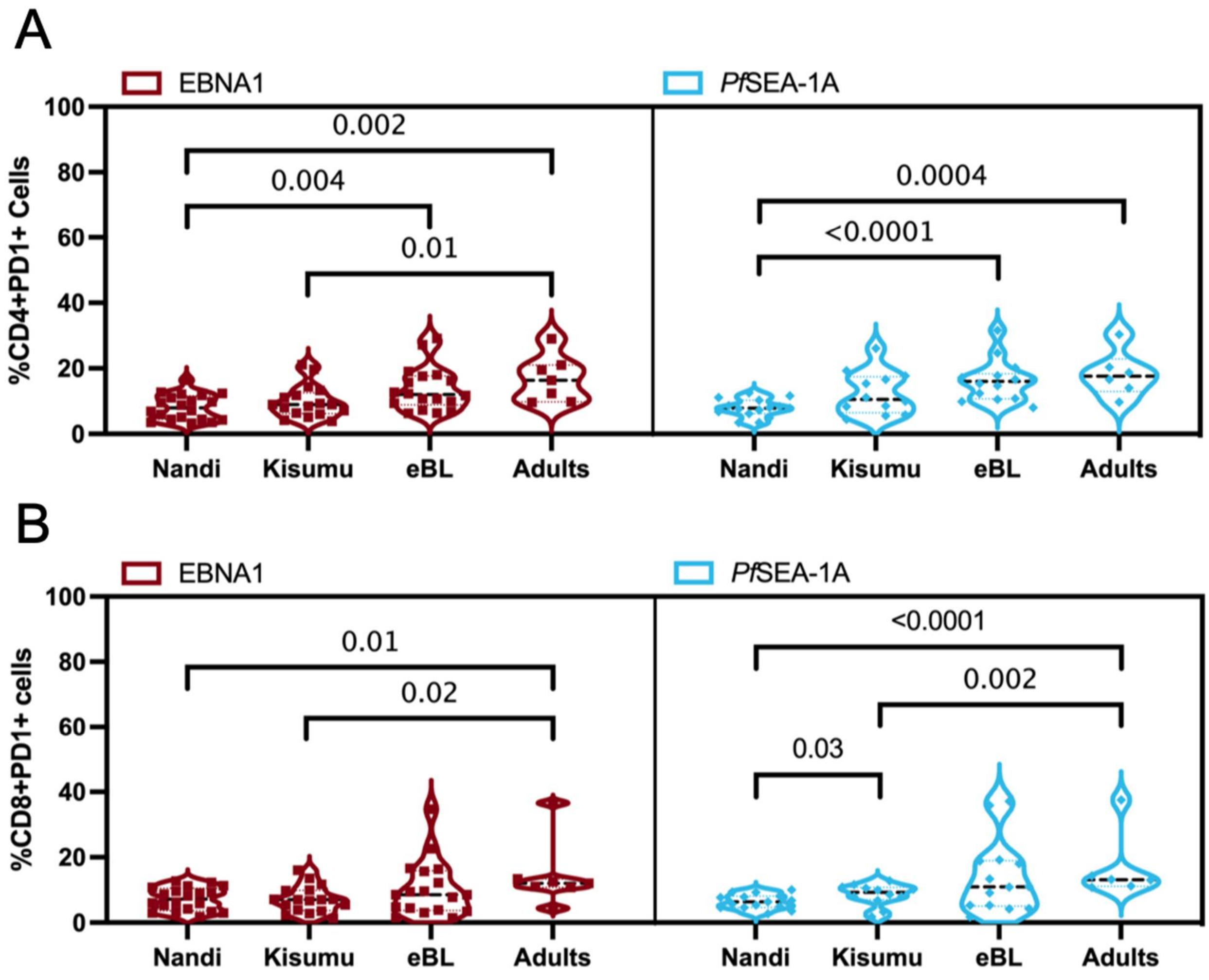

Figure S6. Adults had significantly higher CD4⁺PD-1⁺ T cells compared to Nandi and Kisumu children but not compared to eBL children. (**A**) Percentage of CD4⁺PD-1⁺ cells after EBNA1 (red squares) and *Pf*SEA-1A (blue lozenges) stimulation across groups of individuals. (**B**) Percentage of CD8⁺PD-1⁺ cells after EBNA1 and *Pf*SEA-1A stimulation across groups of individuals. *p* values were calculated using Mann-Whitney test.

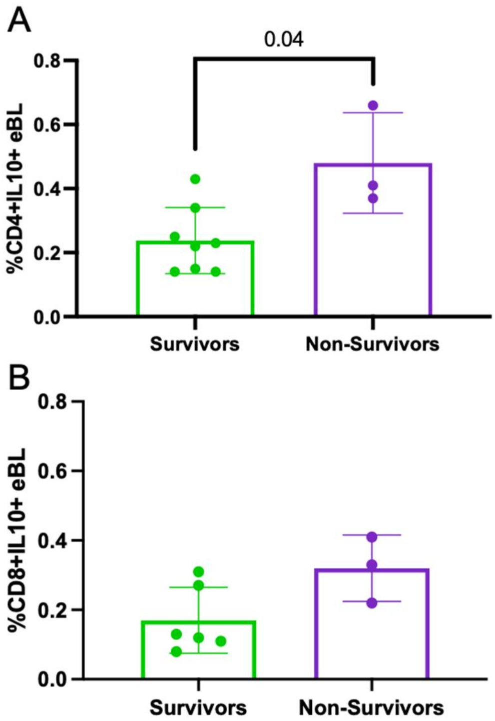

Figure S7. EBNA1-specific IL-10$^+$CD4$^+$ and CD8$^+$ T cell frequencies for eBL survivors compared to non-survivors. (**A**) The percentage of IL-10-expressing CD4$^+$ T cells was significantly higher for eBL non-survivors compared to survivors. (**B**) The percentage of IL-10-expressing CD8$^+$ T cells did not differ by eBL outcome. *p* values were calculated using Mann-Whitney test, mean and SD are represented.
